# Anti-Photoaging Effects of a Standardized Hot Water Extract of *Petasites japonicus* Leaves in Ultraviolet B-Exposed Hairless Mice

**DOI:** 10.3390/ph18101490

**Published:** 2025-10-03

**Authors:** Hyeon-A Song, Min-Ji Park, Chae-Won Lee, Sangsu Park, Jong Kil Lee, Kyung-Sook Chung, Kyung-Tae Lee

**Affiliations:** 1 Department of Pharmaceutical Biochemistry, College of Pharmacy, Kyung Hee University, Seoul 02447, Republic of Korea; 2 Department of Fundamental Pharmaceutical Science, Graduate School, Kyung Hee University, Seoul 02447, Republic of Korea; 3 Naturescience Inc., Neorenaissance Center, Seoul 02447, Republic of Korea; x-zara@nate.com

**Keywords:** photoaging, UVB, collagen, MAPK/AP-1, TGF-β/Smad

## Abstract

**Background:** Ultraviolet B (UVB) radiation accelerates skin aging by inducing oxidative stress, collagen degradation, and cellular senescence. Although *Petasites japonicus* is known for its antioxidant properties, its anti-photoaging potential remains underexplored. This research explored the protective properties of a hot water extract from *P. japonicus* leaves (KP-1) against photoaging caused by UVB exposure. **Methods:** Hairless mice were exposed to UVB three times per week and orally administered KP-1 for 13 weeks. Wrinkle formation, epidermal thickness, skin hydration, and collagen content were assessed. Protein expression related to MAPK/AP-1, TGF-β/Smad2/3, and p53/p21 pathways was analyzed by Western blotting. **Results:** KP-1 significantly reduced UVB-induced wrinkle area, epidermal and dermal thickening, and transepidermal water loss while restoring collagen density and skin hydration. KP-1 inhibited MMP-1 expression, enhanced COL1A1 levels, suppressed MAPK/AP-1 activation, and activated TGF-β/Smad2/3 signaling. It also balanced p53/p21 expression and restored cyclin D1 and CDK4 levels, thereby preventing UVB-induced senescence. **Conclusions:** The findings of this research revealed that KP-1 can serve as a promising natural substance for safeguarding the skin from damage and aging caused by UVB exposure.

## 1. Introduction

The skin acts as a primary barrier, protecting the body from environmental factors such as external irritants, pathogens, and ultraviolet (UV) radiation. This intricate organ consists of the epidermis, the dermis, and subcutaneous fat. The epidermis, primarily composed of keratinocytes, consists of an extracellular matrix that is essential for the skin barrier function. Beneath the epidermis, the dermis is populated by fibroblasts and various matrix components, including collagen and elastin, which maintain the skin’s strength and elasticity [1]. Intrinsic and extrinsic processes are the two categories of skin aging. Intrinsic aging, a natural process influenced by genetic factors, leads to gradual structural and functional changes over time. UVB light from the sun causes photoaging, a type of extrinsic aging. This occurs through repeated exposure that accelerates skin aging by inducing DNA damage, oxidative stress, and inflammatory responses [2]. Although UVB constitutes a smaller proportion of UV radiation than UVA, it induces stronger biological effects by promoting MAPK phosphorylation. This activation suppresses procollagen synthesis and upregulates MMP-1 transcription. Overexpression of MMPs degrades collagen fibers within the extracellular matrix, which weakens the skin’s mechanical and structural integrity [3,4].

UVB-induced photoaging is also closely linked to DNA damage, which triggers the p53/p21 pathway, resulting in cell cycle arrest and apoptosis [5]. As a key tumor suppressor, p53 responds to genotoxic stress by halting the cell cycle and inducing apoptosis, while p21 reinforces G1/S arrest by inhibiting CDK activity, and preventing the proliferation of damaged cells. At the same time, cyclin D1/CDK4, which drive skin cell renewal and repair, are disrupted by UVB exposure, impairing regeneration and accelerating cellular senescence [6,7].

*Petasites japonicus*, commonly known as butterbur, is a perennial plant that is native to East Asia, including Japan, Korea, and China [8]. It is traditionally used in herbal medicine for its therapeutic properties and has been studied for its diverse pharmacological effects, such as antioxidant [9], anti-allergic [10], and tumor-suppressing effects [11]. We previously reported that the *P. japonicus* leaf extract (KP-1) contains 3,4-dicaffeoylquinic, 3,5-dicaffeoylquinic, and 4,5-dicaffeoylquinic acids [12], which exhibit hepatoprotective and antidiabetic activities [13,14]. KP-1 also exerts powerful anti-inflammatory effects by suppressing nitric oxide and prostaglandins [15]. However, the anti-photoaging effect of KP-1 against UVB irradiation remains unknown.

Given the growing demand for natural and safe alternatives to synthetic anti-photoaging agents, clarifying the protective role of KP-1 against UVB-induced skin damage is of great significance. Unlike conventional compounds such as retinoids, which may cause irritation or long-term safety concerns, KP-1 represents a food-derived extract with the potential to combine efficacy and safety [16]. These characteristics highlight its potential as a candidate for cosmeceutical and nutraceutical development. We hypothesized that KP-1 would mitigate UVB-induced photoaging by inhibiting MAPK/AP-1 activation, promoting the TGF-β/Smad2/3 pathway, and regulating the p53/p21-mediated cell cycle pathway, thereby enhancing energy and greater potential to cause skin damage [4].

Excessive UVB exposure disrupts the skin structure, reduces its integrity and strength, and leads to increased water loss, wrinkle formation, dryness, and sagging [17]. UVB radiation induces the generation of reactive oxygen species (ROS), which stimulate receptor tyrosine kinases by suppressing receptor protein tyrosine phosphatases. This stimulation triggers downstream signaling molecules, such as mitogen-activated protein kinases (MAPKs), which subsequently activate the activator protein-1 (AP-1) transcription factor. AP-1 promotes the expression of matrix metalloproteinases (MMPs) and inhibits pro-collagen transcription, resulting in collagen degradation and impaired skin integrity [18]. UVB irradiation promotes the activation of transforming growth factor-beta (TGF-β), which binds to its receptors and initiates downstream signaling by inducing Smad2 collagen deposition and reducing wrinkle formation. To test this hypothesis, we conducted in vivo experiments in UVB-irradiated hairless mice and in vitro studies in UVB-exposed HaCaT cells to investigate the underlying molecular mechanisms.

## 2. Results

### 2.1. Identification of KP-1 by HPLC-UV

**Figure 1:**
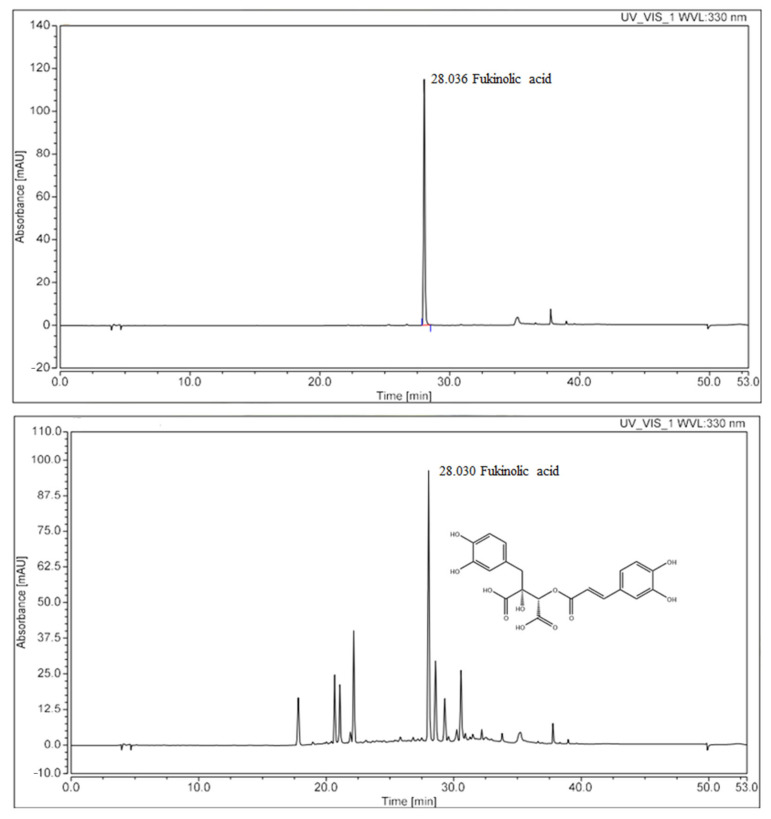
High-Performance Liquid Chromatography (HPLC) result of fukinolic acid and KP-1.

KP-1 chromatogram exhibited a characteristic peak at a retention time of 28.030 min, which was similar to that of the standard, fukinolic acid. This result indicates fukinolic acid as the major component of KP-1 (26.5 mg/g; Figure 1).

### 2.2. KP-1 Inhibits Wrinkle Formation in UVB-Exposed Hairless Mice

**Figure 2:**
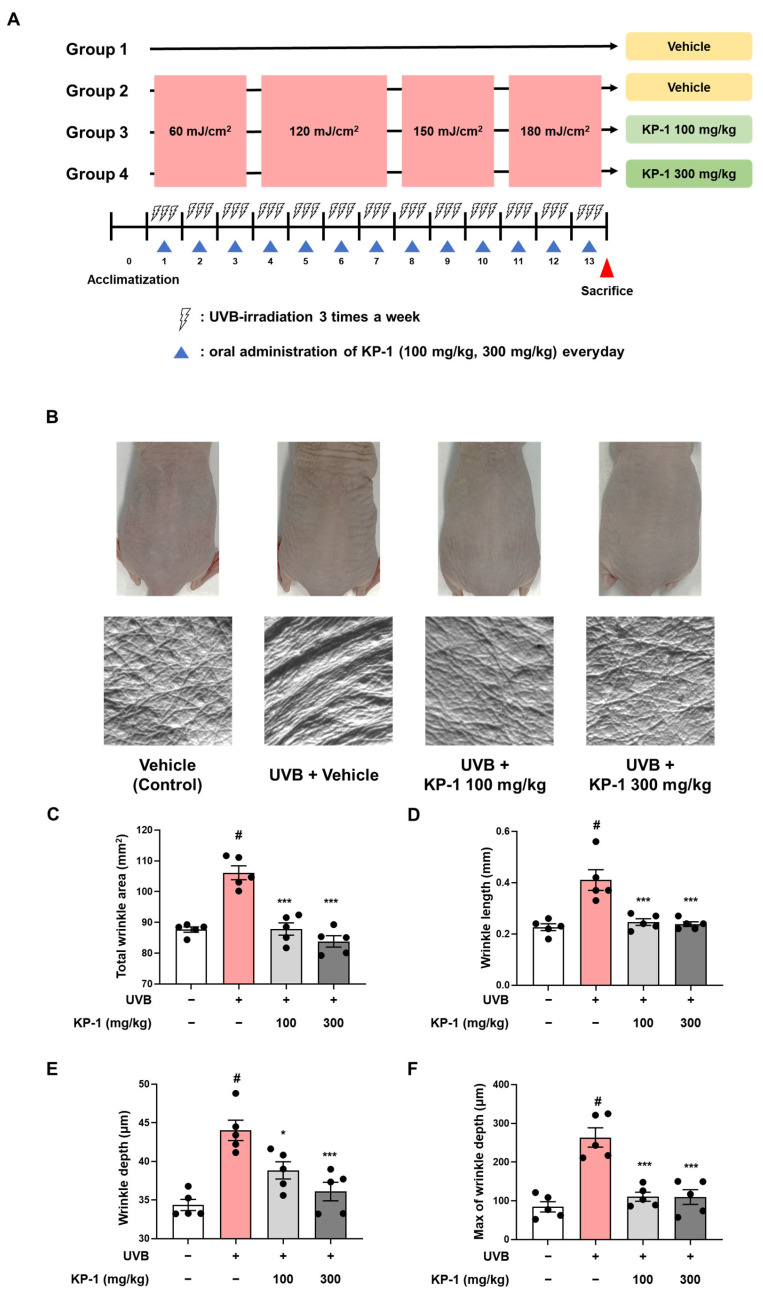
Effect of KP-1 on wrinkle formation in Ultraviolet B (UVB)-irradiated hairless mice. (**A**) Scheme of animal experiments. Hairless mice were administered KP-1 (100 and 300 mg/kg) daily for 13 weeks and simultaneously exposed to UVB (60–180 mJ/cm^2^). (**B**) Images of dorsal and replica skin of mice. Wrinkle parameters: (**C**) total area, (**D**) length, (**E**) depth, and (**F**) maximum depth. Data are presented as the mean ± standard error of the mean (SEM; *n* = 5). *^#^ p* < 0.05 compared with the CON group; * p < 0.05 and *** p < 0.001 compared with the UVB group.

UVB radiation accelerates skin aging by disrupting the extracellular matrix, leading to increased wrinkle formation via the degradation of collagen and elastin fibers [19]. To determine the efficacy of KP-1 in preventing UVB-induced skin damage, hairless mice were exposed to UVB radiation and orally administered KP-1 (100 or 300 mg/kg) for 13 weeks (Figure 2A). As shown in the replica images in Figure 2B, the total wrinkle area significantly increased in UVB-exposed hairless mice compared to that in the non-irradiated controls (CON vs. UVB group: 87.67 ± 0.86 vs. 106.13 ± 2.26 mm^2^; *p* < 0.05). However, KP-1 significantly inhibited wrinkle formation. UVB exposure also significantly increased the wrinkle length (CON vs. UVB group: 0.23 ± 0.01 vs. 0.41 ± 0.04 mm; *p* < 0.05), wrinkle depth (CON vs. UVB group: 34.36 ± 0.72 vs. 44.02 ± 1.32 μm; *p* < 0.05), and maximum wrinkle depth (CON vs. UVB group: 84.27 ± 13.32 vs. 264.35 ± 24.91 μm; *p* < 0.05), whereas KP-1 (100 and 300 mg/kg) significantly (*p* < 0.05) mitigated these effects (Figure 2C–F).

### 2.3. KP-1 Prevents Skin Thickening and Water Loss in Hairless Mice Exposed UVB Irradiation

**Figure 3:**
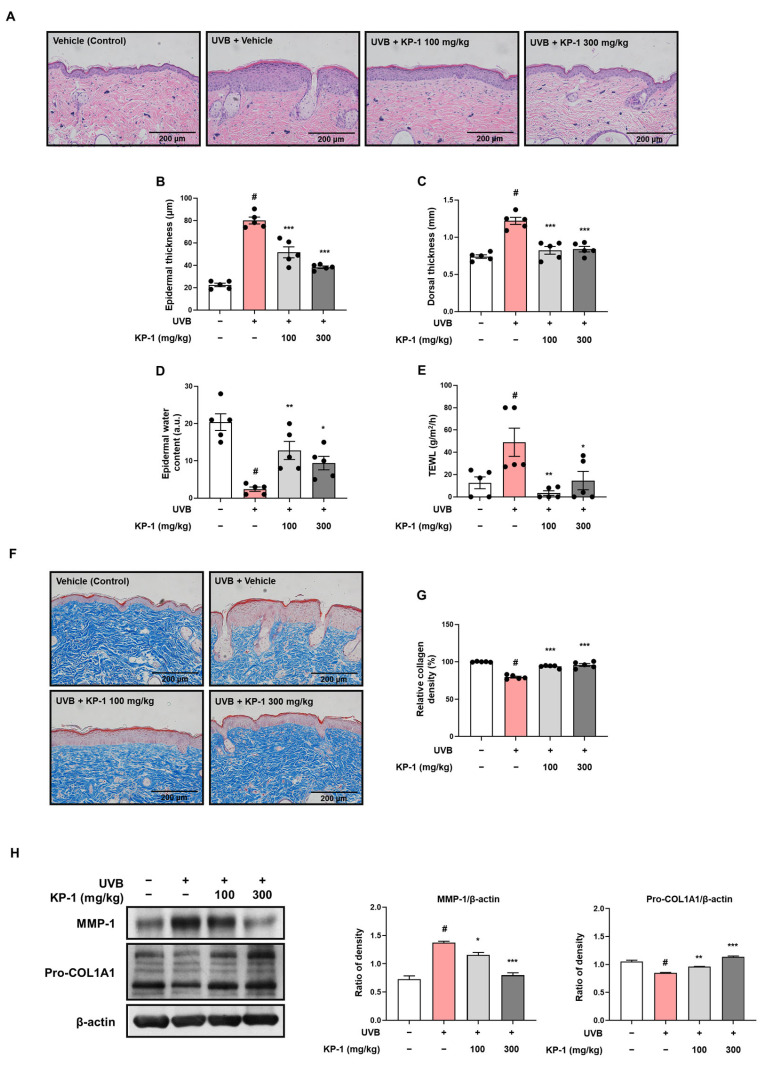
Effects of KP-1 on skin thickening, water content, and collagen degradation in UVB-irradiated hairless mice. (**A**) Hematoxylin and eosin staining analysis of dorsal skin (Scale bars: 200 μm). (**B**) Epidermal thickness. (**C**) Dorsal skin thickness. (**D**) Epidermal water content. (**E**) Transepidermal water loss (TEWL). (**F**,**G**) Collagen density analysis using Masson’s trichrome staining (Scale bars: 200 μm). (**H**) Western blotting was employed to assess protein expression levels, with β-actin serving as the internal control. Data are presented as the mean ± SEM (*n* = 5). *^#^ p* < 0.05 compared with the CON group; * p < 0.05, ** p < 0.01, and *** p < 0.001 compared with the UVB group.

UVB irradiation leads to skin barrier dysfunction and increased water loss by altering the protein expression of involucrin and filaggrin, which are vital for skin integrity [20]. Histological analysis was performed to evaluate the effects of orally administered KP-1 on UVB irradiation-induced epidermal thickness and structural integrity of the skin in hairless mice. H&E staining of the vehicle group revealed normal skin histology with a thin epidermis and an intact structure (Figure 3A). In contrast, the UVB group exhibited UVB-induced epidermal hyperplasia and a disrupted dermal architecture. However, treatment with KP-1 (100 and 300 mg/kg) reduced the epidermal thickness and improved the dermal structure compared with those in the UVB group. Epidermal thickness was also measured at random locations in the histological images. UVB group exhibited significantly increased epidermal thickness (CON vs. UVB group: 22.48 ± 1.55 vs. 80.07 ± 3.03 μm; *p* < 0.05), which was notably reduced by KP-1 (KP-1 100 mg/kg group: 51.60 ± 4.88 μm and KP-1 300 mg/kg group: 38.32 ± 1.08 μm; *p* < 0.001; Figure 3B). As shown in Figure 3C, KP-1 decreased the dorsal skin thickness (KP-1 100 mg/kg group: 0.82 ± 0.05 mm and KP-1 300 mg/kg group: 0.84 ± 0.04 mm; *p* < 0.001) increased by UVB exposure (CON vs. UVB group: 0.74 ± 0.02 vs. 1.22 ± 0.05 mm; *p* < 0.05). Epidermal water content and TEWL were measured prior to euthanasia to determine the effects of KP-1 on skin moisturization. UVB irradiation reduced the epidermal water content (CON vs. UVB group: 20.4 ± 2.23 vs. 2.4 ± 0.6 a.u.; *p* < 0.05) compared to that in the CON group, whereas KP-1 reversed this effect (KP-1 100 mg/kg group: 12.8 ± 2.44 a.u. and KP-1 300 mg/kg group: 9.4 ± 1.81 a.u.; *p* < 0.05; Figure 3D). Additionally, KP-1 significantly restored TEWL (KP-1 100 mg/kg group: 3.4 ± 2.09 g/m^2^/h and KP-1 300 mg/kg group: 14.6 ± 8.20 g/m^2^/h; *p* < 0.05) decreased by UVB irradiation (CON vs. UVB group: 12.6 ± 5.27 vs. 49.00 ± 12.66 g/m^2^/h; *p* < 0.05; Figure 3E).

### 2.4. KP-1 Inhibits Collagen Degradation in the Dorsal Skin of UVB-Irradiated Hairless Mice

Collagen, a vital protein in the extracellular matrix of the skin, plays fundamental roles in maintaining the structure, elasticity, and strength of the skin, which are crucial for overall skin health and prevent sagging [21]. Here, we assessed the effect of KP-1 on UVB irradiation-induced collagen degradation using Masson’s trichrome staining in hairless mice. The CON group exhibited a dense and well-organized collagen structure (Figure 3F). In contrast, the UVB group exhibited collagen degradation and disorganization, indicating UVB-induced damage. However, KP-1 preserved the collagen structure compared with that in the UVB group. The quantitative analysis results of collagen are shown in Figure 3G. The UVB group exhibited a significantly decreased collagen content (CON vs. UVB group: 100 ± 0.23% vs. 79.48 ± 1.22%; *p* < 0.05), which was significantly restored by KP-1 (KP-1 100 mg/kg group: 93.70 ± 0.87% and KP-1 300 mg/kg group: 95.77 ± 1.88%; *p* < 0.001). To further evaluate whether KP-1 inhibited collagen degradation, we performed Western blotting. As shown in Figure 3H, UVB irradiation increased MMP-1 expression and decreased collagen type I α 1 chain (COL1A1) expression; however, KP-1 effectively reversed these effects.

### 2.5. KP-1 Modulates the Activation of the MAPK/AP-1 and TGF-β/Smad2/3 Pathways Triggered by UVB

**Figure 4:**
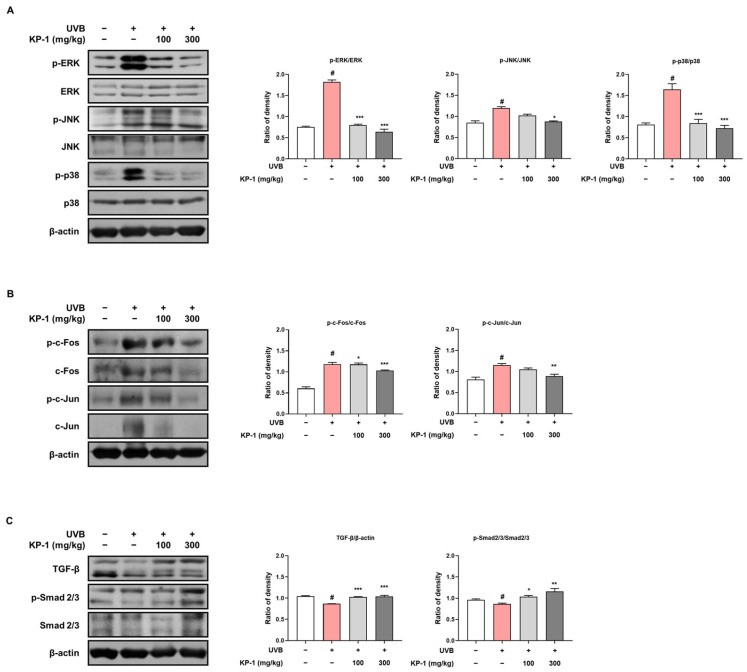
Effects of KP-1 on the mitogen-activated protein kinase (MAPK)/activator protein (AP)-1 and transforming growth factor (TGF)-β/Smad 2/3 signaling pathways in UVB-irradiated hairless mice. Protein expression levels of (**A**) MAPKs, (**B**) AP-1, and (**C**) TGF-β/Smad 2/3 were assessed by Western blotting. β-actin is used as an internal control. Data are presented as the mean ± SEM (*n* = 5). *^#^ p* < 0.05 compared with the CON group; * p < 0.05, ** p < 0.01, and *** p < 0.001 compared with the UVB group.

MAPK signaling plays a crucial role in maintaining the protective barrier of the skin by suppressing TGF-β signaling, a key regulator of procollagen type I synthesis [22]. Here, UVB exposure increased MAPK phosphorylation, whereas KP-1 effectively reversed this effect and inhibited the phosphorylation of ERK, JNK, and p38 in the dorsal skin of hairless mice (Figure 4A). Additionally, KP-1 decreased the phosphorylated c-Fos and c-Jun levels to the control levels (Figure 4B). KP-1 markedly upregulated TGF-β protein expression and promoted Smad2/3 phosphorylation in the 100 and 300 mg/kg groups (Figure 4C).

### 2.6. KP-1 Regulates UVB-Induced Cell Cycle Alterations

**Figure 5:**
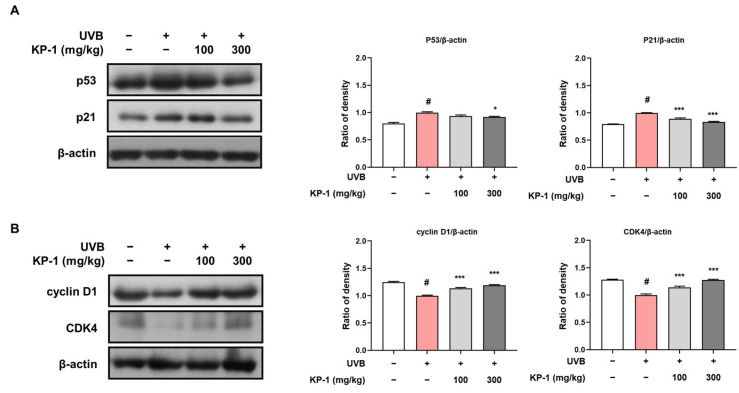
Effects of KP-1 on cell cycle regulation in UVB-irradiated hairless mice. Western blotting was employed to determine the expression levels of proteins (**A**) p53 and p21, along with (**B**) cyclin D1 and CDK4 with β-actin serving as the internal control. β-actin was used as the internal control. Data are presented as the mean ± SEM (*n* = 5). *^#^ p* < 0.05 compared with the CON group; * p < 0.05 and *** p < 0.001 compared with the UVB group.

UVB-induced cell cycle dysregulation triggers cellular senescence and accelerates photoaging in the skin [23]. In this study, we evaluated the dorsal skin of UVB-irradiated hairless mice for expression levels of key cell cycle regulators, such as p53, p21, cyclin D1, and CDK4. UVB exposure led to a notable increase in the levels of the cell cycle inhibitors p53 and p21 compared with those in the control group (Figure 5A). KP-1 treatment effectively suppressed this UVB-induced upregulation, resulting in reduced levels of p53 and p21 in both KP-1-treated groups. Moreover, UVB exposure resulted in a reduction in cyclin D1 and CDK4 expression, which are essential for cell cycle progression (Figure 5B). KP-1 treatment restored cyclin D1 and CDK4 expression to levels comparable to those in the control group. These results demonstrate that KP-1 regulates both negative and positive cell cycle regulators, thereby contributing to the maintenance of cellular homeostasis under UVB-induced stress.

## 3. Discussion

UV-induced photoaging is characterized by collagen degradation, wrinkle formation, and impaired skin barrier function [24]. While synthetic compounds such as retinoids and alpha-hydroxy acids are commonly used to counteract these effects, their application is often limited by adverse reactions, including skin irritation and photosensitivity [25]. Given the increasing interest in safer alternatives, plant-derived compounds warrant further investigation. Therefore, continued research on natural bioactive compounds with minimal side effects is essential for the development of safer and more effective anti-photoaging interventions.

*P. japonicus*, a member of the *Asteraceae* family, is a herbaceous perennial plant distributed across East Asia. It possesses medicinal properties, exerts potent anti-inflammatory effects, and alleviates memory impairment [26,27]. Fukinolic acid-containing *P. japonicus* exerts antioxidant effects to mitigate oxidative stress, which is a key inducer of photoaging [28]. Based on these antioxidant properties, we speculated that KP-1 could be effective against photoaging. However, as a plant extract, KP-1 contains multiple bioactive compounds. While this study primarily focused on its overall efficacy, further research is needed to elucidate the individual contributions of its active components.

KP-1 is considered safe for oral administration, as it has already been reported to show no oral toxicity, 13-week repeated oral toxicity, or genotoxicity [12]. The anti-photoaging effects of KP-1 in UVB-exposed hairless mice were evaluated by analyzing dorsal skin wrinkle formation and epidermal thickness, along with the associated molecular pathways. The safety of orally administered KP-1 was evaluated by determining plasma levels of glutamic oxaloacetic transaminase (GOT), glutamic pyruvic transaminase (GPT), and blood urea nitrogen (BUN) in this mouse model (Figure S1). In the UVB group, GOT and GPT levels were significantly increased compared with those in the CON group, indicating UVB irradiation induced hepatotoxic stress. In contrast, KP-1 administration did not alter GOT, GPT, or BUN levels relative to those in the CON group, suggesting that KP-1 did not cause hepatotoxicity or nephrotoxicity. Given that *P. japonicus* has long been consumed as food without notable adverse effects, this background further supports the favorable safety profile of KP-1 and suggests its potential suitability for long-term application.

The skin, composed of the epidermis, dermis, and hypodermis, undergoes significant changes upon UVB exposure, which primarily affects the epidermis, causing sunburn, wrinkle formation, and photoaging [29]. In our study, KP-1 treatment visibly reduced wrinkle formation and roughness on the dorsal skin compared to the UVB group. To objectively measure wrinkle parameters, we assessed the overall area, length, depth, and maximum depth of wrinkles using samples obtained from the dorsal skin of mice. Administration of KP-1 significantly restored wrinkle parameters induced by UVB irradiation. Upon UVB exposure, the epidermis undergoes hyperplasia, which increases the number of keratinocyte layers as a defense mechanism to provide an additional barrier against further damage. This hyperplastic response increases epidermal thickness and disrupts the integrity of the stratum corneum, leading to increased TEWL and decreased skin hydration [30]. Consistent with these observations, UVB irradiation-induced epidermal and dorsal thickening, reduced the water content, and increased TEWL. In contrast, the KP-1-administered group showed reduced thickness and dehydration, suggesting that KP-1 exerts photoprotective effects.

UV radiation contributes to skin aging by generating ROS, which trigger the skin aging pathways. ROS produced by UVB exposure further upregulates MMPs, especially MMP-1, leading to the breakdown of the collagen framework in skin tissues [31]. Collagen type I, the most abundant collagen in the skin, is crucial for skin structural integrity and primarily responsible for maintaining the tensile strength, resilience, elasticity, and overall strength of the skin [32]. As *P. japonicus* exerts antioxidant effects [28], we investigated whether KP-1 inhibits collagen degradation in UVB-irradiated hairless cells. We found that KP-1 suppressed the expression of MMP-1. Additionally, KP-1 increased the collagen density and expression of pro-COL1A1, the precursor of type I collagen. To further investigate whether the observed effects of KP-1 in vivo were associated with its regulation of collagen synthesis and degradation, we conducted in vitro experiments using UVB-irradiated HaCaT cells. The results showed that KP-1 treatment increased the mRNA expression of COL1A1 while suppressing *MMP-1* expression, indicating its potential role in promoting collagen synthesis and inhibiting its breakdown at the transcriptional level (Supplementary Figure S2).

UVB exposure triggers the MAPK and AP-1 signaling pathways, leading to inflammation and skin damage [33]. This process begins with UVB-induced generation of ROS, which activates the MAPK pathways, such as the ERK, JNK, and p38 pathways [34]. These kinases further stimulate the AP-1 transcription factor, contributing to the production of proinflammatory cytokines and MMPs [35]. In this study, KP-1 reduced UVB-induced phosphorylation of MAPK (ERK, JNK, and p38) and AP-1 (c-Fos and c-Jun) components, thereby decreasing inflammation and cellular damage. Similarly, Zhang reported that *Prunella vulgaris* extract exerts protective effects via the NF-kB, MAPK, AP-1, and TGF-β/Smad signaling pathways in UVB-aged normal human dermal fibroblasts [36]. Furthermore, KP-1 enhanced the levels of TGF-β and phosphorylation of Smad2/3, which are crucial for collagen synthesis and repair. The TGF-β/Smad signaling pathway is activated when TGF-β attaches to its receptor, causing Smad2/3 phosphorylation, translocation to the nucleus, and the promotion of procollagen synthesis [37].

To gain further insight into the inhibitory effects of KP-1 on MAPK activation, we examined the protein expression levels of phosphorylated JNK and p38 in UVB-irradiated HaCaT cells (Supplementary Figure S3). KP-1 treatment significantly reduced the phosphorylation of JNK and p38 while maintaining total JNK and p38 levels, supporting its role in suppressing MAPK activation in response to UVB exposure.

UVB exposure disrupts cell cycle regulation, leading to growth arrest, cellular senescence, and impaired proliferation, all of which contribute to skin aging. In response to UVB-induced stress, p53 and p21 are upregulated in response to UVB-induced stress, triggering G1 arrest for DNA repair, but prolonged activation accelerates senescence. Meanwhile, UVB reduces cyclin D1 and CDK4 expression, hindering cell cycle progression and skin regeneration [37,38]. In this study, KP-1 helped to counteract these effects by decreasing UVB-induced p53 and p21 expression, preventing excessive growth arrest and cellular senescence. Additionally, KP-1 restored cyclin D1 and CDK4 levels, promoting cell cycle progression and supporting skin regeneration. These findings suggest that KP-1 plays a key role in maintaining cellular homeostasis, mitigating UVB-induced senescence, and contributing to its anti-photoaging effects. Given these protective effects against UVB, KP-1 may also exhibit potential efficacy against other UV wavelengths, such as UVA and UVC. Further studies are necessary to investigate its broader photoprotective properties and underlying mechanisms.

Our results demonstrate that KP-1 mitigates UVB-induced photoaging by modulating key molecular pathways. Specifically, KP-1 inhibited MAPK/AP-1 activation, enhanced TGF-β/Smad2/3 signaling, and regulated p53/p21-mediated cell cycle arrest, contributing to collagen preservation and wrinkle reduction. These effects suggest that KP-1 not only reduces inflammation and oxidative stress but also supports cellular homeostasis and regeneration. These findings reject the null hypothesis and support the hypothesis that KP-1 exerts its anti-photoaging effects by modulating key molecular pathways. Beyond these experimental outcomes, KP-1 may also be applied to human skincare as a functional cosmetic ingredient, with further potential in cosmeceutical and nutraceutical applications aimed at preventing photoaging and improving skin health.

Although each experimental group consisted of only five mice and no positive control compound was included, this study consistently demonstrated the anti-photoaging efficacy of KP-1. Nevertheless, these limitations highlight the need for future investigations with larger sample sizes and well-established reference agents to strengthen the translational relevance of our findings. Taken together, KP-1 emerges as a promising anti-photoaging agent that targets both inflammatory responses and collagen degradation, thereby contributing to skin elasticity and wrinkle reduction.

## 4. Materials and Methods

### 4.1. Preparation and Analysis of KP-1 Using High-Performance Liquid Chromatography (HPLC)

KP-1 was prepared according to a previously reported protocol [12]. KP-1 was filtered through a membrane syringe filter (0.45 μm, Agilent Technologies, Santa Clara, CA, USA) before injecting 10 μL. Fukinolic acid was dissolved in formic acid–methanol–water (0.1–50–49.9% *v*/*v*/*v*). The standard solutions were prepared stepwise to a concentration range of 12.5 to 100 µg/mL. High-performance liquid chromatography (HPLC) was performed using a Thermo Ulti-Mate 3000 system (Thermo Fisher Scientific, Waltham, MA, USA) consisting of an UltiMate 3000 pump, an UltiMate 3000 autosampler, and an UltiMate 3000 variable wavelength detector. Chromatograms were processed using Chromeleon Dionex software, Version 7.2.10.23925. The Discovery C18 analytical column (250 × 4.6 mm, 5 µm pore size, Supelco, Bellefonte, PA, USA) was filled with the same stationary phase. Linear gradient elution of eluents A (0.1% *v/v* aqueous formic acid) and B (0.1% *v/v* formic acid in acetonitrile) was used for separation. The gradient program included the following phases: 0–10 min, 5–5% B; 10–15 min, 5–15% B; 15–20 min, 15–20% B; 20–25 min, 20–25% B; 25–30 min, 25–40% B; 30–35 min, 40–95% B; 35–45 min, 95–95% B; 45–47 min, 95–5% B; and the last one was 47–53 min, 5–5% B. The column temperature was set at 25 °C, the UV detector was adjusted to 330 nm, and the flow rate was kept at 0.8 mL/min.

### 4.2. Ethical Statement

All animal experiments were performed in full compliance with the regulations set by the Kyung Hee University Animal Care and Use Committee, which reviewed and approved the study protocol (Approval No. KHSASP-22-536) on 26 October 2022. At the end of the experiment, euthanasia was performed using a CO_2_ chamber, ensuring complete loss of consciousness before confirming death by cessation of heartbeat and respiratory movement. All procedures were supervised by trained personnel to ensure ethical compliance.

### 4.3. Experimental Animals and Sample Treatment

Five-week-old male hairless mice were sourced from SLC Inc. (Shizuoka, Japan) and maintained in a controlled environment with a temperature of 22 ± 1 °C and humidity levels of 50 ± 10%, following a 12 h light/dark cycle. The mice had unrestricted access to food and water. Following a one-week period for acclimatization, the animals were divided into four groups at random (*n* = 5/group): a control group treated with vehicle (CON group), UVB + vehicle-treated group (UVB group), and UVB + KP-1-treated groups (100 or 300 mg/kg. p.o.). KP-1 was dissolved in distilled water and administered orally every day. UVB irradiation was performed using a UVP Crosslinker (Analytik Jena AG, Jena, Germany). The UVB exposure protocol for the treated groups involved irradiation three times weekly for 13 weeks, with doses increasing as follows: 1–3 weeks, 60 mJ/cm^2^; 4–7 weeks, 120 mJ/cm^2^; 8–10 weeks, 150 mJ/cm^2^; and 11–13 weeks, 180 mJ/cm^2^.

### 4.4. Skin Wrinkle Formation Analysis

At the conclusion of the experiment, replicas of dorsal skin were made from the hairless mice using the SILFLO kit (Monaderm, 5 Rue des Violettes, Monaco) to assess wrinkle formation. Wrinkle parameters were analyzed according to the methods described in our previous report [39].

### 4.5. Histological Analysis

Dorsal skin tissues were embedded in paraffin, sectioned, and stained with hematoxylin and eosin (H&E) for examining skin layer alterations. Epidermal thickness was determined by calculating the average thickness in three different locations of each section. Masson’s trichrome staining was performed to analyze collagen fiber density, which was measured using the ImageJ software (version 1.54m).

### 4.6. Measurement of the Thickness of Dorsal Skin, the Water Content in the Epidermis, and the Loss of Water Through the Epidermis (TEWL)

The thickness of the dorsal skin was assessed with a digital caliper. The GPSkin Barrier^®^ device (GPOWER Inc., Seoul, Republic of Korea) was used to assess the water content and transepidermal water loss (TEWL) of the dorsal skin.

### 4.7. Western Blot Analysis

Proteins from dorsal skin tissues were extracted using PRO-PREP solution (Intron Biotechnology, Seoul, Republic of Korea). Quantified proteins (25 μg) were separated on 8–10% SDS-PAGE gels and transferred onto PVDF membranes. Membranes were incubated with primary antibodies in 5% skim milk at 4 °C for 24 h. Following this, the membranes were incubated with secondary antibodies in 5% skim milk at 25 °C for 2 h. Detection of immune blots was achieved using enhanced chemiluminescence (ECL) substrates. The antibodies employed are detailed in Table S1.

### 4.8. Statistical Analysis

The null hypothesis (H_0_) stated that KP-1 treatment does not significantly affect collagen deposition, MAPK/AP-1 activation, or TGF-β/Smad2/3 signaling in UVB-irradiated skin. Conversely, the alternative hypothesis (H_1_) proposed that KP-1 exerts significant protective effects against UVB-induced photoaging through modulation of these pathways.

To test this hypothesis, all data are presented as the mean ± standard error of the mean (SEM, *n* = 5). The experimental findings were analyzed statistically using GraphPad Prism software (version 8.0.2; GraphPad Software Inc., San Diego, CA, USA). To determine significance, a one-way ANOVA was employed. A *p*-value below 0.05 was deemed to indicate statistical significance.

## 5. Conclusions

In this study, we demonstrated the protective effects of KP-1 against UVB-induced skin damage in hairless mice. KP-1 treatment alleviated wrinkle formation, reduced epidermal thickness, and preserved collagen structure. Additionally, KP-1 modulated key signaling pathways, including MAPK/AP-1 and TGF-β/Smad2/3, and maintained p53/p21 activity within a balanced range to prevent excessive cell cycle arrest. By employing both in vivo and in vitro approaches, this work provides comprehensive evidence that a standardized extract of *P. japonicus* can counteract photoaging through multiple mechanisms. These findings suggest that KP-1 may be further explored as a promising candidate for human skincare, with potential applications in cosmeceutical and nutraceutical development.

## Data Availability

The original contributions presented in this study are included in the article/Supplementary Material. Further inquiries can be directed to the corresponding author(s).

## Supplementary Materials

The following supporting information can be downloaded at: https://www.mdpi.com/article/10.3390/ph18101490/s1, Table S1: List of antibodies used in the experiment, Table S2: Primer sequences used in qRT-PCR, Figure S1: Effects of KP-1 on the plasma level of GOT, GPT, and BUN, production in the UVB-irradiated hairless mice, Figure S2: Effects of KP-1 on the mRNA expression levels of COL1A1 and MMP-1 in UVB-irradiated HaCaT cells, Figure S3: Effects of KP-1 on the mitogen-activated protein kinase (MAPK) pathway in UVB-irradiated HaCaT cells.
