# Supplementary material for: Anti-Photoaging Effects of a Standardized Hot Water Extract of Petasites japonicus Leaves in Ultraviolet B-Exposed Hairless Mice
Source: Pharmaceuticals (Basel). 2025 Oct 3;18(10):1490. doi: 10.3390/ph18101490 (PMC12567178; doi:10.3390/ph18101490)
Supplement: Supplementary file 1 [file pharmaceuticals-18-01490-s001.zip › pharmaceuticals-3840172-supplementary.pdf]

Supplementary Data

Supplementary methods

Measurement of glutamic oxaloacetic transaminase (GOT), glutamic pyruvic transaminase (GPT), and blood urea nitrogen (BUN) in the UVB-irradiated hairless mice.

Whole blood samples (approximately 300 μL per mouse) were gathered using tubes coated with heparin, from which plasma was separated for the biochemical analysis, including glutamic oxaloacetic transaminase (GOT), glutamic pyruvic transaminase (GPT), and blood urea nitrogen (BUN). The procedures for preparing and conducting the biochemical test of plasma were followed by the guidelines of T&P Bio Co. (Gwangju, Republic of Korea).

In Vitro UVB irradiation and KP-1 treatment

HaCaT cells, a human keratinocyte cell line, were obtained from ATCC (Manassas, VA, USA) and cultured according to ATCC's guidelines. Cells were seeded at a density of 5×10^5 cells/mL in culture plates and allowed to adhere overnight. The next day, cells were washed with phosphate-buffered saline (PBS) and subjected to UVB irradiation (15 mJ/cm^2) using a UVP Crosslinker CL-1000M (Analytik Jena AG, Jena, Germany). After UVB exposure, the cells were treated with KP-1 at concentrations of 30, 100, and 300 μg/ml in DMEM and incubated under standard conditions.

RNA Extraction and Quantitative Real-Time RT-PCR (qRT-PCR)

Total RNA was isolated from cells using Easy Blue® kits (Intron Biotechnology, Seoul, Republic of Korea). A total of 500 ng of extracted mRNA was reverse transcribed into cDNA using random oligonucleotide primers (Promega, WI, USA) and TOPscript™ RT DryMIX (Enzynomics, Daejeon, Republic of Korea). The expression levels of target genes were

quantified via real-time PCR using the QuantStudio 1 system (Thermo Fisher Scientific, Waltham, USA) with specific primers listed in Table S2.

Supplementary Table S1. List of antibodies used in the experiment.

Supplier	Location	Antibodies
Santa Cruz Biotechnology Inc.	Dallas, TX, USA	pro-COL1A1 (sc-25973), ERK (sc-93), JNK (sc-7345), c-Fos (sc-253), p53 (sc-6243), p21 (sc-817), CDK4 (sc-260), cyclin D1 (sc-8396), β-actin (sc-81178)
Cell Signaling Technology Inc.	Danvers, MA, USA	p-ERK (#4377), p-JNK (#4668), p-p38 (#9215), p-38 (#9212), p-c-Fos (#5348), TGF-β (#3711), p-Smad 2/3 (#8828), Smad 2/3 (#5678)
GeneTex	Irvine, CA, USA	MMP-1 (GTX100534)

Supplementary Table S2. Primer sequences used in qRT-PCR

Gene	Forward Sequence	Reverse Sequence
hCOL1A1.	AGGGCCAAGACGAAGACATC	AGATCACGTCATCGCACAACA
hMMP-1	GCCCAGGTATTGGAGGGGAT	AGGGTACATCAAAGCCCCGA
hGAPDH	TCAAGTGGGGCGATGCTG	ACCCATGACGAACATGGGG

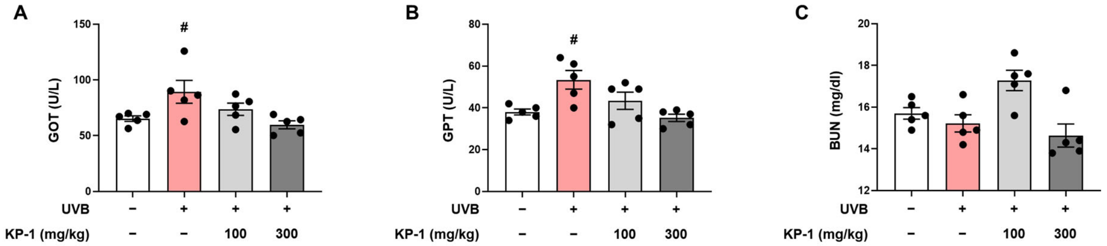

Supplementary Figure S1. Effects of KP-1 on the plasma level of GOT, GPT, and BUN, production in the UVB-irradiated hairless mice. The production of (A) GOT, (B) GPT, and (C) BUN were determined by the ELISA kit. Data are represented as the mean ± SEM (n = 5). $^{\#}p < 0.05$ compared with the CON group.

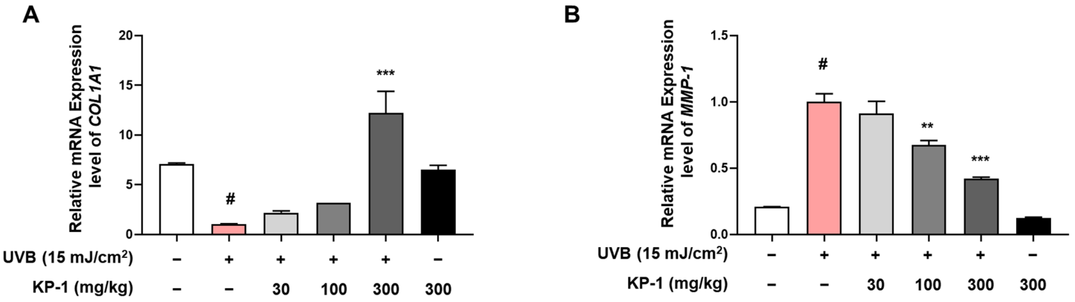

Supplementary Figure S2. Effects of KP-1 on the mRNA expression levels of *COL1A1* and *MMP-1* in UVB-irradiated HaCaT cells. The mRNA expression levels of (A) COL1A1 and (B) MMP-1 were assessed by qRT-PCR. Data are represented as the mean ± SD (n = 3). $^{\#}p < 0.05$ compared with the CON group; $^{*}p < 0.05$, $^{**}p < 0.01$, and $^{***}p < 0.001$ compared with the UVB group.

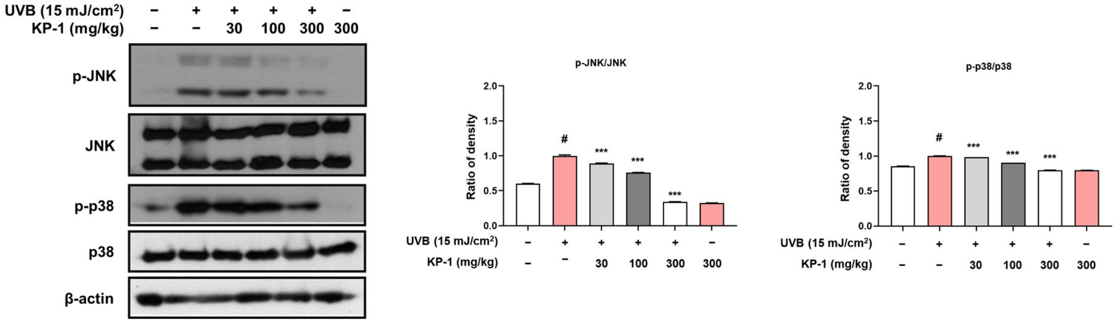

Supplementary Figure S3. Effects of KP-1 on the mitogen-activated protein kinase (MAPK) pathway in UVB-irradiated HaCaT cells. Protein expression levels of MAPKs were assessed by western blotting. β-actin is used as an internal control. Data are represented as the mean ± SD (n = 3). $^{\#}p < 0.05$ compared with the CON group; $^{*}p < 0.05$, $^{**}p < 0.01$, and $^{***}p < 0.001$ compared with the UVB group.
